# A Case Report of Granulomatosis with Polyangiitis Presenting with Chronic Conjunctivitis

**DOI:** 10.5070/M5.52284

**Published:** 2026-07-31

**Authors:** Daniel Martin

**Affiliations:** * University of Texas, Southwestern Medical Center, Department of Emergency Medicine, Dallas, TX

## Abstract

An adult female presents for chronic eye complaints refractory to outpatient ophthalmology management, as well as purpuric rash, Reynaud’s symptoms, and reported nodules. She received ophthalmology and rheumatology consultations while in the emergency department (ED) and was subsequently admitted. Expedited rheumatologic workup revealed evidence of granulomatosis with polyangiitis, a rare and potentially debilitating autoimmune vasculitis. She was ultimately discharged after an eight-day hospital course on a long prednisone taper with plan for outpatient management and transition to long-term therapy. This report documents a case warranting high clinical suspicion for an undiagnosed condition in a patient with demonstrably poor access to longitudinal care and the subsequent, in-hospital diagnosis of a rare disease facilitated by ED clinician action.

**Topics:**

Granulomatosis with polyangiitis, vasculitis, autoimmune, ophthalmology, rash.

**Figure:**
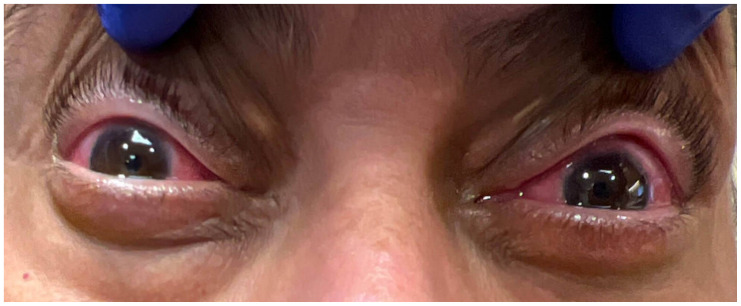


**Figure:**
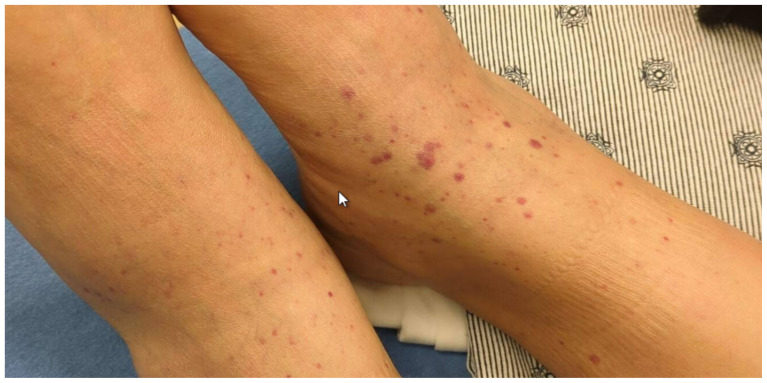


## Brief introduction

Granulomatosis with polyangiitis (GPA) is the most common anti-neutrophil cytoplasmic antibodies (ANCA)-associated vasculitis^1^,^2^. It is distinguishable from its related eosinophilic variant, EGPA or Churg-Strauss syndrome, and microscopic polyangiitis (MPA) by current diagnostic standards.^1^–^5^ Notably, GPA was previously known as Wegener’s Granulomatosis, but its eponymous designation was formally abandoned by the multiple international societies in the early 2000’s in favor of disease-descriptive nomenclature.^2^ Though ANCA positivity is a hallmark of GPA (>90% positivity), several other rheumatologic factors are also believed to be involved in the inflammatory disease cascade which results in the characteristic pathologic changes of GPA.^1^–^3^,^5^–^8^ These include anti-proteinase 3 (anti-PR3) and anti-myeloperoxidase (anti-MPO) with varying respective rates of positivity and theorized mechanisms of action.^1^,^2^,^5^–^8^ Several genetic associations have also been investigated and described in the literature, including a defective alpha-1 antitrypsin allele.^6^ Certain environmental exposures, including commonly used antibiotics, allopurinol, and hydralazine have also been associated with ANCA-associated vasculitis.^4^ As with most rheumatologic conditions, the causal factors and associations are multi-factorial, imperfectly understood, and difficult to fully characterize for any given patient.

The pathophysiology of GPA, as the name describes, involves immune-mediated vasculitis of small and medium-sized vessels and granulomas secondary to neutrophilic microabscesses.^9^ The resulting vasculitis often affects the upper respiratory tract (clinical complaint in 90% of cases), lower respiratory tract (50% with pulmonary infiltrates), glomerulonephritis (develops in 80% of cases within two years of onset), eye involvement (various clinical complications in >50% of cases), lower extremity purpura, neuropathies, arthralgias, and myalgias.^7^,^8^,^10^–^13^ GPA primary affects adults rather than children, and the comparably infrequent pediatric cases have generally similar presentations excluding a higher incidence of subglottic stenosis.^11^,^14^,^15^

## Presenting concerns and clinical findings

A 55-year-old Hispanic female with prior diagnoses of dacryoadenitis, type 2 diabetes mellitus (T2DM), and gastroesophageal reflux presented for evaluation of bilateral eye redness for multiple months, intermittent palpable nodules on her hands, dacryoadenitis, and a purpuric macular rash on bilateral ankles for the last two weeks. On chart review, it is noted that she has several presentations over three years to the ED for eye redness sometimes associated with headache, as well as various polyarthralgias, sinusitis, and pharyngitis. Prior exams had consistently noted bilateral proptosis, and at least one prior consult by the ophthalmologists had noted elevated intra-ocular pressure (IOP) resulting in pressure-lowering medications. CT orbits showed enlarged lacrimal glands, and an eventual surgical orbitotomy with biopsy of a lacrimal gland was performed. The biopsy was reported as negative for IG4, the apparent diagnosis applied was dacryoadenitis, and she had ultimately been lost to ophthalmologic follow up not long after that procedure. This biopsy had occurred two years prior to her current presentation, and another CT brain performed due to an unrelated trauma since then had not revealed any differing finding. Her last two ED presentations were within four months of this presentation for eye complaints and headaches. She reports that at various times she has received steroids which seemed to improve her condition for a time. She was not currently using any steroids or immunomodulatory medications.

## Significant findings

The patient’s vital signs were stable, with HR 92, BP: 139/76, Temp: 36.4 °C, RR: 17, SpO2: 100% on room air. Physical exam revealed an adult woman who was alert, oriented, pleasantly conversational, and in no apparent acute distress. Notable bilateral proptosis with conjunctival injection and limbic sparing (clear conjunctiva surrounding the margin of the cornea, shown by the arrow in the image of the eyes) was present. Visual acuity was 20/70 OS and 20/20 OD. There was a purpuric macular rash on her bilateral ankles. Her reported intermittent palpable nodules were not currently present, nor was an active episode of Reynaud’s phenomenon apparent. Written consent was obtained for use of patient images in this case report.

## Patient course

The primary differential diagnoses included viral or irritant conjunctivitis, seasonal environmental allergies, rheumatologic or other vasculitis. Less likely diagnoses considered included occlusive vascular diseases or other chronic ophthalmic conditions. Basic labs revealed mild hypochromic, microcytic anemia, thrombocytosis, normal blood creatinine and eGFR, urinalysis showing glucose >1000mg/dl, 3+ blood, protein 100mg/dl, urobilinogen 2mg/dl, leukocyte esterase 250 leu/mcl. Tonometry values were within normal limits. Given her persistent and progressive ocular symptoms, ophthalmology was consulted and noted non-specific inflammatory changes on their slit lamp exam with no retinal pathology. They recommended magnetic resonance imaging (MRI) orbits with and without contrast and otherwise concurred with consult to rheumatology given her history and other symptoms. In consultation with rheumatology, additional labs were ordered which were notable for elevated CRP 18.5mg/dl (normal 0.5), ESR 103mm/hr (normal 1–19), abnormal c-ANCA 1:160 (normal <1:20), negative ANA, elevated C3 Complement 287mg/dl (normal 90–180) and C4 Complement 44mg/dl (normal 10–40), significantly elevated anti-proteinase 3 antibody >8.0 units (normal 0.0–0.9), and significantly elevated rheumatoid factor (RF) of 83.0IU/ml (normal <14.0).

Magnetic resonance imaging of the brain and orbit/face/neck with and without contrast was obtained which revealed “Bilateral left greater than right enlargement of the lacrimal glands with mild edema of the left supraorbital soft tissues. Findings may represent lacrimal gland infection, inflammation, or dacryoadenitis. Differential diagnosis is broad and would also include etiologies including sarcoid, lymphoma, orbital pseudotumor/IgG4 related disease, inflammatory disease such as Sjogren’s syndrome.” Transthoracic echo performed by the ED clinician revealed no obvious vegetations or other pathologic findings.

Due to the patient’s poor access to outpatient care and repeated presentations to the ED, she was ultimately admitted for expedited workup and treatment. A diagnosis of presumed GPA was made based on her clinical picture and her returned rheumatologic laboratory workup. She remained hospitalized for a total of eight days. A regimen of subcutaneous insulin, basal plus sliding scale, was initiated for management of her blood glucose on day one. She became persistently tachycardic and febrile between days two and three, triggering antipyretics and blood cultures which were ultimately negative. On day four, rheumatology recommended prednisone 60mg daily pending additional labs, and they further recommended a chest computed tomography (CT) and renal biopsy. Nephrology discussed renal biopsy with her, and she declined. The CT Chest showed diffuse ground glass opacities. On day six, she was started on pulse steroids with IV methylprednisolone 1g daily for three days and trimethoprim-sulfamethoxazole for pneumocystis jirovecii prophylaxis. By day seven, her polyarthralgias had resolved and her conjunctival injection was notably improved. Additionally, she completed her pulse steroids and received a first dose of retuximab.

The patient was well-served by her hospital course because she left with significant improvement to her symptoms and a meaningful diagnosis for her thus-far-mysterious clinical presentation. It is likely that, without the additional effort to involve rheumatology from the ED, she would have suffered many more months of poorly treated symptoms and possibly presented to the ED on additional dates while awaiting outpatient workup by the rheumatologists. She was discharged on day eight on a 22-day prednisone taper, continued trimethoprim-sulfamethoxazole, PO iron for her iron-deficiency anemia, insulin for T2DM, and outpatient clinic follow-up referrals to rheumatology, renal, ophthalmology, and primary care. Since her discharge, documentation of one follow-up appointment with ophthalmology indicates their plan is to periodically follow up on ophthalmic symptoms of the patient’s GPA which they expect to be primarily managed by rheumatology.

## Discussion

Granulomatosis with polyangiitis is an insidious condition with variable presentation and is frequently subject to significant delays in diagnosis, especially in patients who have poor access to longitudinal outpatient care and management^1^–^3^,^5^,^9^. Non-specific symptoms, which may suggest autoimmune or vasculitic conditions, such as persistent conjunctivitis, Reynaud’s phenomenon, purpuric lower limb rashes, and end organ damage such as nephropathies should raise suspicion in the emergency clinician for GPA^1^,^2^,^4^,^7^,^9^,^11^,^13^–^17^. The long-term sequelae of untreated GPA make awareness and suspicion for the diagnosis important for emergency clinicians to consider. Long-term management of GPA primarily involves glucocorticoids, biologics, and other disease-modifying anti-rheumatic drugs.^1^–^3^,^7^,^14^,^16^ Plasmapheresis may be indicated in rare cases.^11^,^14^ Management of complications involves supportive care for the affected system, such as dialysis for ESRD.^7^,^17^

Diagnosis can be difficult, especially in the ED setting. One set of diagnostic criteria for GPA are described by the 2022 American College of Rheumatology/European Alliance of Associations for Rheumatology (ACR/EULAR) Classification Criteria for Granulomatosis with Polyangiitis.^2^ For patients in whom small- or medium-vessel vasculitis has been identified, these criteria define the diagnosis using a scoring system which includes physical exam findings, imaging, and laboratory results, though these are not authoritative since they have reported sensitivity of 93% (95% confidence interval [95% CI] 87–96%) and the specificity was 94% (95% CI 89–97%).^2^ This case report adds to a relatively small base of literature concerning presentations of undiagnosed GPA to the ED in which a patient whose chronic ophthalmologic complaints eluded meaningful diagnosis and management over multiple ED presentations and outpatient ophthalmology management.^7^,^13^ When she presented again with persistent and progressive symptoms which were classically associated with autoimmune and vasculitic conditions, the ED clinicians recognized an opportunity to appropriately leverage available resources and admit for expedited rheumatologic workup.^7^,^13^ The definitive diagnosis of GPA, including the distinguishing of GPA from other vasculitic conditions, is not generally going to be completed in the ED. Use of the ACR/EULAR Criteria alone may not have raised significant suspicion in the ED for GPA given her lacking several of the expected positive findings and their lacking description of the nevertheless common ophthalmologic complications.^1^,^2^,^13^ The emergency clinician should recognize chronic ocular symptoms as potentially indicative of GPA because evidence suggests they are present in up to half of GPA patients^3^,^7^,^13^ Presence of an anterior scleritis capable of causing blindness, uveitis, ulcerative keratitis, and retrobulbar pseudotumor causing proptosis are well-described.^7^,^12^,^13^ Various dermatologic manifestations have been described in roughly half of patients, including purpuric rashes commonly in the lower limbs.^7^ A number of scoring systems and similar clinical tools have been developed; none of these tools are validated for assessment of undifferentiated patients and diagnosis of GPA in the ED.^1^,^2^ Laboratory workup is complex, including ANCA and other serologies, all of which have varying rates of positivity in known GPA cases and none of which represent a definitive diagnostic test alone.^1^–^3^,^6^,^8^,^18^ Though minimum standards of care might have been satisfied with minimal emergency room workup for a patient whose chronic complaint had already been previously evaluated extensively by specialists without improvement, the extra attention she received on this visit yielded a high-value and rare diagnosis. For clinicians who may encounter similar instances of ophthalmic complaints with other associated symptoms, this case should encourage them to consider GPA and leverage appropriate hospital resources or make appropriate outpatient referrals with appropriate communication to the patient.^12^,^13^

## Supplementary Information




